# The effect of everolimus and low-dose cyclophosphamide on immune cell subsets in patients with metastatic renal cell carcinoma: results from a phase I clinical trial

**DOI:** 10.1007/s00262-018-2288-8

**Published:** 2019-01-17

**Authors:** Charlotte M. Huijts, Sinéad M. Lougheed, Zuhir Bodalal, Carla M. van Herpen, Paul Hamberg, Metin Tascilar, John B. Haanen, Henk M. Verheul, Tanja D. de Gruijl, Hans J. van der Vliet, for the Dutch WIN-O Consortium

**Affiliations:** 1 Department of Medical Oncology, Amsterdam UMC, Vrije Universiteit Amsterdam, Cancer Center Amsterdam, De Boelelaan 1117, 1081 HV Amsterdam, The Netherlands; 2 Department of Medical Oncology, Radboud University Medical Center, Nijmegen, The Netherlands; 3 Department of Medical Oncology, Franciscus Gasthuis and Vlietland, Rotterdam, The Netherlands; 4 Department of Medical Oncology, Isala Clinics, Zwolle, The Netherlands; 5 Division of Medical Oncology, Netherlands Cancer Institute, Amsterdam, The Netherlands

**Keywords:** Everolimus, Cyclophosphamide, mRCC, Tregs, mTOR, Immune monitoring

## Abstract

For the treatment of metastatic renal cell cancer several strategies are used among which the mTOR inhibitor everolimus. As mTOR plays an important role in the immune system, e.g., by controlling the expression of the transcription factor FoxP3 thereby regulating regulatory T cells (Tregs), it plays a key role in the balance between tolerance and inflammation. Previous reports showed stimulatory effects of mTOR inhibition on the expansion of Tregs, an effect that can be considered detrimental in terms of cancer control. Since metronomic cyclophosphamide (CTX) was shown to selectively deplete Tregs, a phase 1 clinical trial was conducted to comprehensively investigate the immune-modulating effects of several dosages and schedules of CTX in combination with the standard dose of everolimus, with the explicit aim to achieve selective Treg depletion. Our data show that 50 mg of CTX once daily and continuously administered, in combination with the standard dose of 10 mg everolimus once daily, not only results in depletion of Tregs, but also leads to a reduction in MDSC, a sustained level of the CD8^+^ T-cell population accompanied by an increased effector to suppressor ratio, and reversal of negative effects on three peripheral blood DC subsets. These positive effects on the immune response may contribute to improved survival, and therefore this combination therapy is further evaluated in a phase II clinical trial.

**Electronic supplementary material:**

The online version of this article (10.1007/s00262-018-2288-8) contains supplementary material, which is available to authorized users.

## Introduction

Kidney cancer is one of the ten most common cancer types in both men and women, with an estimated number of 338,000 new cases per year [1]. The most common tumor arising in the kidney is renal cell carcinoma (RCC) and a total of more than ten different subtypes can be identified [2]. The therapeutic field of metastatic RCC (mRCC) has drastically changed in the past decade with the introduction of the VEGF signaling pathway inhibitors and inhibitors of mammalian target of rapamycin (mTOR) [3]. For years, everolimus has been the standard second-line treatment after a VEGF-based treatment regimen until the arrival of axitinib as an alternative and recently nivolumab and cabozantinib, drugs that inhibit the PD-1 immune checkpoint and the MET, AXL and VEGF tyro-sine kinases, were shown to be more effective compared to everolimus [4–6]. Furthermore, the progression-free survival (PFS) of patients with mRCC was improved by addition of the multi-target tyrosine kinase inhibitor lenvatinib to everolimus [7].

Everolimus has been shown to be an effective inhibitor of mTOR, resulting in inhibition of cell growth, proliferation, angiogenesis and survival of tumor cells [8]. However, mTOR also plays an important role in the regulation of the immune response, by promoting the expansion of regulatory T cells (Tregs) [9, 10]. Since Tregs have immune suppressive capacities, this Treg-promoting effect of everolimus can be considered a detrimental effect in the treatment of cancer. In support of this notion, increased Treg numbers have been associated with poor survival in patients with cancer, including mRCC [11–13].

Several strategies have been investigated to selectively deplete Tregs, among them the use of low-dose cyclophosphamide (CTX). Administration of metronomic low-dose CTX was reported to selectively deplete Tregs, with additional beneficial effects on T and NK cell functionality [14, 15]. Therefore, a phase 1 clinical trial was initiated to prevent everolimus-induced detrimental Treg expansion, by adding metronomic CTX to the standard dosage of everolimus [16], to achieve improved survival by modulating the immune system. Patients were treated in cohorts of five patients, with six different doses and schedules of CTX. Clinical results and results of changes in Treg frequencies in the various cohorts of this phase 1 trial were separately described [17]. Here, we report on the results of the extensive and comprehensive immune monitoring that was additionally performed in this phase 1 study, where patients were treated with either everolimus alone or the combination of everolimus and different CTX administration dosages and schedules.

## Materials and methods

### Study population

Forty patients with mRCC and previously treated with a VEGF targeting regimen were treated with everolimus in combination with different doses and schedules of metronomic oral CTX. Thirty-nine patients were evaluable, since one patient was not able to complete 2 weeks of the treatment due to early toxicity. The trial was initiated by the department of medical oncology of the Amsterdam UMC, location VUmc and conducted within the context of the Netherlands Working Group on Immunotherapy of Oncology (WIN-O) with participation of 13 hospitals and enrollment of patients from January 2012 until August 2015. Clinical findings were reported separately [17].

### Treatment

Patients were treated with a fixed dose of 10 mg everolimus once daily and enrolled in one of the seven cohorts, five patients per cohort, with different doses and schedules of low-dose oral CTX. One patient in dose level 6 stopped treatment because of several toxicities (highest grade 3 nausea) within 2 weeks of enrollment and was not evaluable. CTX was scheduled week on/week off or continuously, once or twice daily, based on the previously used dose regimens reported by Ghiringhelli et al. [15]. In cohort 0, patients were treated with 10 mg everolimus without CTX. In cohort 1, patients were treated with everolimus and 50 mg CTX once daily, week on/week off. In cohort 2, patients were treated with everolimus and 50 mg CTX once daily in a continuous scheme. In cohort 3, patients received 50 mg CTX twice daily, week on/week off, and in cohort 4 patients received 50 mg CTX twice daily, continuously. In the last two cohorts, cohort 5 and 6, respectively, patients received 100 mg CTX twice daily, in cohort 5 in a week on/week off regimen and in cohort 6 continuously.

### Immune monitoring

At baseline and after 2, 4, and 8 weeks after the start of study treatment, 60 mL of heparinized peripheral blood was collected for immune monitoring. All materials were processed on the same day the blood was drawn. PBMC were isolated by density-gradient centrifugation with Lymphoprep (Axis-Shield, Oslo, Norway). After isolation, PBMC were stored overnight at 4 °C in RPMI 1640 (Lonza, Basel, Switzerland) supplemented with 100 IU/ml sodium penicillin (Astellas Pharma, Leiden, the Netherlands), 100 mg/ml streptomycin sulfate (Radiumfarma-Fisiofarma, Naples, Italy), 2.0 nM L-glutamine (Life Technologies, Bleiswijk, the Netherlands), 10% FBS (HyClone, Amsterdam, the Netherlands), and 0.05 mM 2-ME (Merck, Darmstadt, Germany). The next day, cells were stained for flow cytometric analysis.

### Flow cytometry

FITC, PE, PerCP or allophycocyanin (APC)-labeled antibodies directed against human CD3, CD4, CD8, CD11c, CD14, CD16, CD19, CD25, CD56, CD86, CD123, CTLA-4, HLA-DR, Ki-67, PD-1, (all BD Biosciences, New Jersey, USA), CD33, (Beckman Coulter Inc., California, USA), CD56 (IQ Products, Groningen, the Netherlands), and blood DC antigens BDCA1, BDCA2, BDCA3 (all from Miltenyi Biotec, Bergisch-Gladbach, Germany) and matching isotype control antibodies were used. Stainings were performed in PBS supplemented with 0.1% BSA and 0.02% sodium azide for 30 min. Intracellular staining was performed after fixation and permeabilization using a fixation/permeabilization kit according to the manufacturer’s protocol (eBioscience). For staining of FoxP3, a PE-labeled Ab against FoxP3 (clone PCH101, eBioscience) or AlexaFLuor488 FoxP3 (clone 259D) (Biolegend) was used. Live cells were gated based on forward and side scatter and analyzed on a BD FACSCalibur (BD Biosciences) and analyzed using Kaluza Analysis Software (Beckman Coulter).

### Statistical analysis

One-way repeated measures ANOVA was used to determine the statistical significance of differences within cohorts with Dunnett’s multiple comparison test as post-test. Two-way ANOVA was used to compare the mean values between cohorts. Differences were considered statistically significant when *p* values were ≤ 0.05, as indicated with asterisks (*p ≤ 0.05, **p < 0.01, ***p < 0.001). Statistical analyses were performed using GraphPad Prism software (version 7, 2016).

## Results

### The addition of a once daily oral dose of 50 mg CTX to treatment with everolimus results in Treg depletion and an increase in the CD8^+^ T cell: Treg ratio without changes in T-cell activation

**Fig. 1:**
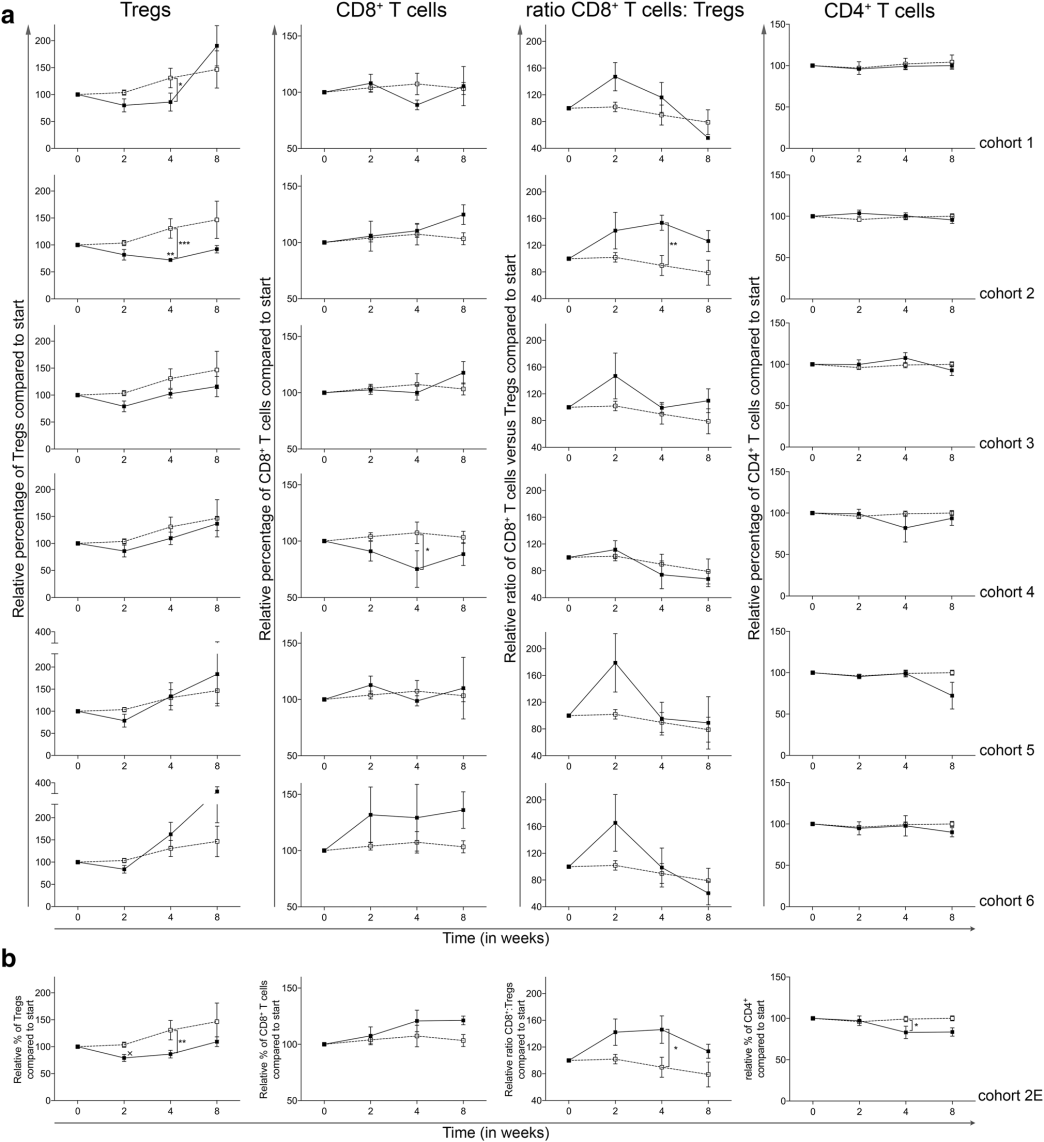
Effect of different dosages and administration schedules of CTX when combined with a fixed dose of 10 mg everolimus on the frequency of Tregs, CD8^+^ T cells, the effector to suppressor (CD8:Treg) ratio and CD4^+^ T cells. **a** Relative percentages (to start) of Tregs, CD8^+^ T cells, the effector to suppressor ratio and CD4^+^ T cells were determined in freshly isolated PBMC from patients treated with different dosages and schedules of CTX, combined with a fixed dose of everolimus at baseline and subsequently 2, 4, and 8 weeks after start of treatment. Cohorts 1–6 correspond to the different CTX dosages and schedules investigated (black bullets, black line) and are compared to cohort 0, the everolimus only cohort (open bullet, dotted line). Tregs were determined within CD4^+^ T cells, CD8^+^ T cells and CD4^+^ T cells within CD3^+^ T cells. **b** Relative percentages of Tregs, CD8^+^ T cells, the effector to suppressor ratio and CD4^+^ T cells are shown for the expansion cohort. Patients were again treated with 50 mg CTX once daily, combined with 10 mg everolimus once daily as previously in cohort 2. Means ± SEM are shown

As previously reported [16], the main objective of this trial was to determine the optimal dose and schedule of orally administered CTX, when combined with 10 mg everolimus, to obtain selective Treg depletion. As shown in Fig. 1a (left graphs), cohort 2, the cohort where 10 mg everolimus was combined with 50 mg CTX continuously, showed a significant decrease in Treg percentages (within CD4^+^ T cells), both within the cohort, comparing the percentages at time point 0 to time point 4, and compared to the corresponding time point 4 in cohort 0, the everolimus only cohort, whereas CD4^+^ T-cell percentages remained stable (Fig. 1a and Supplementary Table 1). Cohort 2 was the only cohort in which this effect was observed. Except for cohort 4, in which a significant decrease in CD8^+^ T cells was observed in comparison to cohort 0 at time point 4, no major differences were observed between cohorts in CD8^+^ T-cell frequencies. On the other hand, the ratio of CD8^+^ T cells to Tregs was significantly increased in cohort 2 compared to cohort 0 at week 4 (Fig. 1a). This increase in CD8^+^ T cell:Treg ratio was only statistically significant in cohort 2. Based on the Treg-depleting data in cohort 2 and the observation that the Treg-depleting effect of CTX was less pronounced in subsequent cohorts, with even an increase in Treg percentages in cohort 5 and 6 (see Fig. 1a), the decision was made to proceed to the expansion cohort wherein an additional 5 patients were treated with the combination of 10 mg everolimus and 50 mg CTX continuously (as in cohort 2). The expansion cohort again showed a significant decrease in Treg percentages at time point 4 in comparison to Treg percentages from cohort 0 and a significant increase in the CD8^+^ T cell:Treg ratio, thereby confirming the previously observed results of cohort 2 (Fig. 1b).

For T-cell activation, PD-1 and CTLA-4 expression was determined on CD4^+^ and CD8^+^ T cells. Overall, no consistent or persistent changes in either PD-1 or CTLA-4 expression on either subset of T cells could be observed (Supplementary Fig. 1a). This was also the case for cohort 2 and the expansion cohort 2E (Supplementary Fig. 1b). As Supplementary Fig. 1 shows relative values, absolute percentages of PD-1 and CTLA-4 expression on CD4^+^ and CD8^+^ T cells are shown in supplementary table 2.

As a measure of the proliferative activity of Tregs and CD4^+^ T cells, Ki-67 expression was determined in both cell types. As shown in Supplementary Fig. 2, a significant decrease in Treg Ki-67 expression was observed within cohort 3 and 5 comparing the expression at baseline to time point 2. In addition, the percentage of Ki-67^+^ Tregs in cohort 5 was significantly increased compared to cohort 0 at week 4 (Supplementary Fig. 2a, left panels) and a similar trend (not significant) was observed in cohort 6. For the CD4^+^ T cells, a significantly lower percentage of cells expressed Ki-67 at week 2 in cohort 2 as compared to week 0 (*p* < 0.001). Although not significant, an increase in CD4^+^ T cells expressing Ki-67 was seen at time point 4 for cohort 2 and subsequent cohorts showed a similar increase at time point 4, with a significant effect in cohort 6.

The results of the expansion cohort 2E (Supplementary Fig. 2b) were similar to cohort 2, however, the Ki-67^+^ Tregs in the expansion cohort first showed a significant decrease at week 2, followed by a significant increase at week 4. Furthermore, the increase in Ki-67^+^ Treg cells at time point 4 was more abundant and significantly different compared to cohort 0. Although an increase in Ki-67 expression in CD4^+^ T cells was also observed, it failed to reach statistical significance.

### CTX results in a decrease in the frequency of monocytic MDSC

**Fig. 2:**
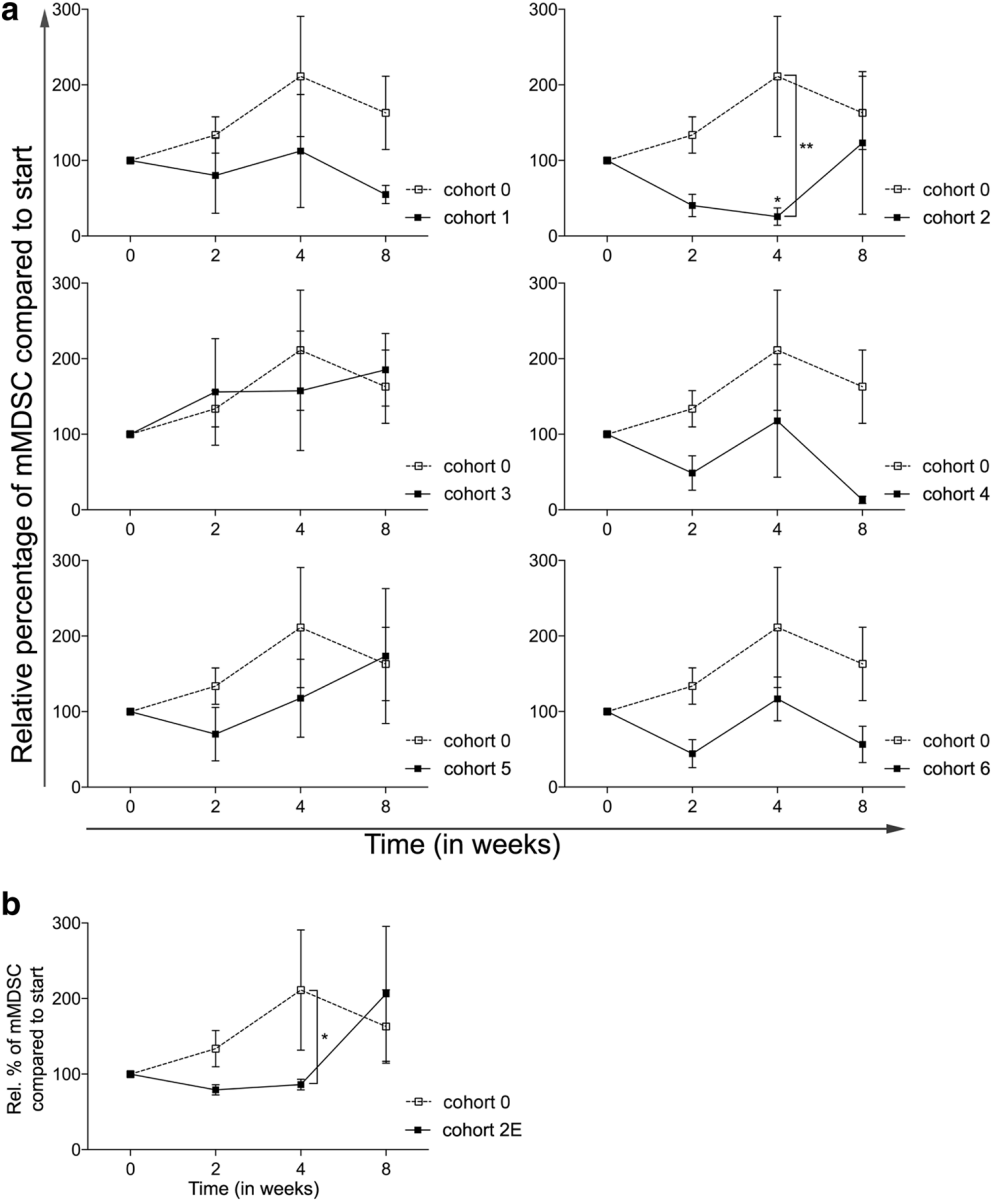
Effect of different dosages and administration schedules of CTX when combined with a fixed dose of 10 mg everolimus on the frequency of mMDSC. **a** Relative percentages of mMDSC (to start) defined as Lin^−^CD14^+^HLA-DR^−^ are shown for the six investigated CTX cohorts (black bullets, black line), compared to cohort 0 (open bullet, dotted line). **b** Relative percentages of mMDSC are shown for the expansion cohort. Means ± SEM are shown

As MDSC are key players in immune suppression in the tumor microenvironment as well as systemically, being able to contribute to tumor progression and metastasis [18], the percentages of monocytic MDSC (mMDSC, defined as Lin^−^CD14^+^HLA-DR^−^) in peripheral blood were determined in all cohorts. As shown in Fig. 2a, treatment with everolimus alone resulted in a non-significant increase in mMDSC. With the exception of cohort 3, addition of CTX resulted in a decrease in the frequency of mMDSC. In cohort 2, this decrease relative to levels in cohort 0 reached statistical significance (*p* < 0.01) at time point 4 weeks. In addition, within cohort 2, a significant difference between mMDSC percentages at baseline versus time point 4 was observed (*p* < 0.05). Although less pronounced, the results of the expansion cohort confirmed the earlier observed decrease in the frequency of mMDSC, with a significant difference between the expansion cohort and cohort 0 at time point 4 (*p* < 0.05, Fig. 2b).

### Addition of CTX reverses the effects of everolimus on blood DC subsets

**Fig. 3:**
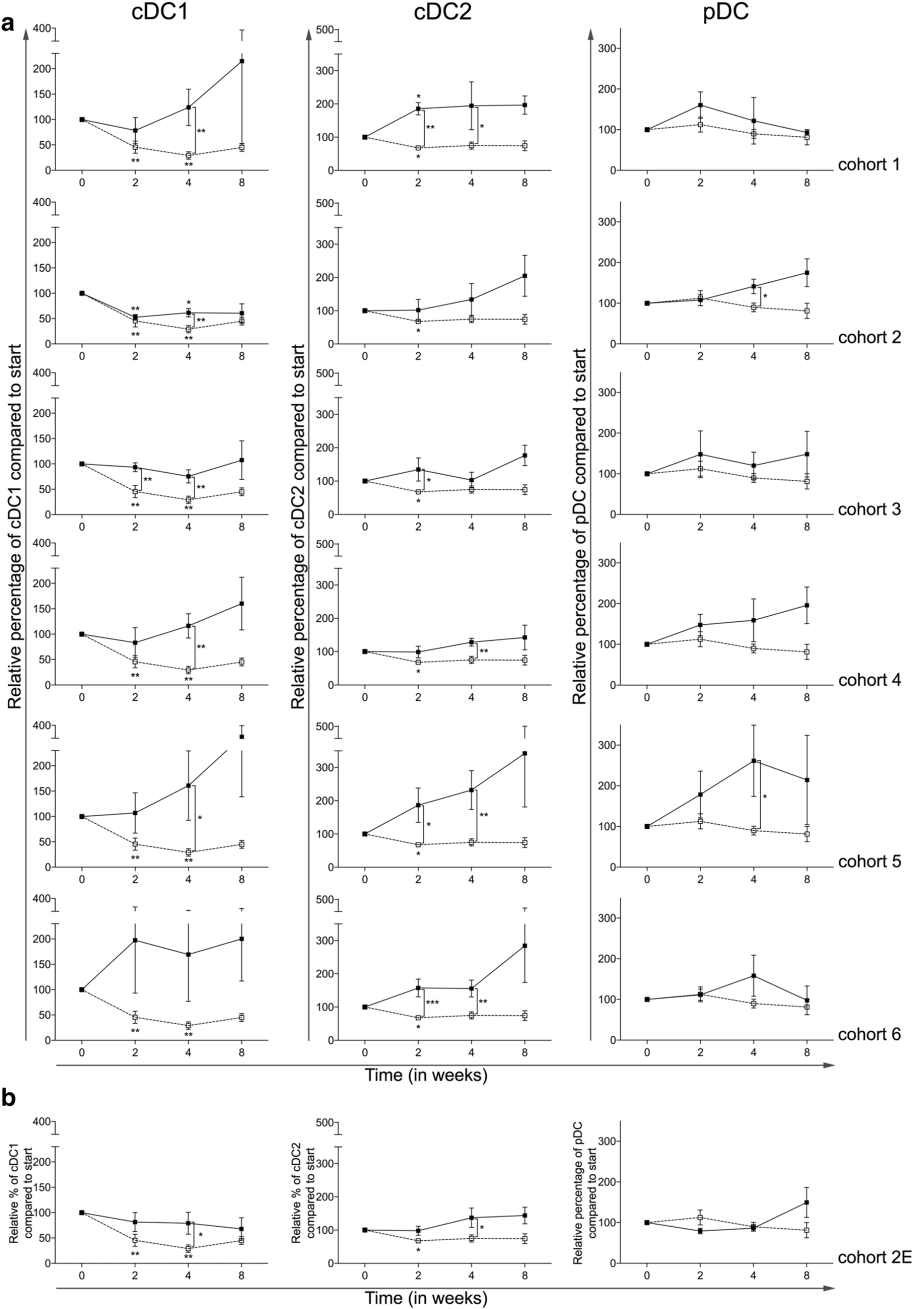
Effect of different dosages and administration schedules of CTX when combined with a fixed dose of 10 mg everolimus on the frequency of three blood DC subsets. **a** Relative percentages of cDC1 (BDCA3^+^CD14^−^CD11c^+^), cDC2 (BDCA1^+^CD19^−^CD14^−^CD11c^+^), and pDC (BDCA2^+^CD123^+^) are shown for the six investigated CTX cohorts (black bullets, black line), compared to cohort 0 (open bullet, dotted line), relative to start. **b** Relative percentages of the three subsets are shown for the expansion cohort. Means ± SEM are shown

To assess the effects of the combination of everolimus and CTX on blood DC subsets, the percentages and activation status of three blood DC subsets were determined, i.e., conventional DC1 (cDC1, defined as BDCA3^+^CD14^−^CD11c^+^), cDC2 (defined as BDCA1^+^CD19^−^CD14^−^CD11c^+^), and plasmacytoid DC (pDC, defined as BDCA2^+^CD123^+^) [19]. The activation status of these subsets was determined by MFI measurement of CD86 (and in addition CD40, data not shown). In cohort 0, a significant decrease in the frequency of cDC1 at time point 2 and 4 weeks, and of the cDC2 subset at time point 2 weeks was noted. Addition of CTX diminished these effects (Fig. 3a). Interestingly, an actual increase in both cDC1 and cDC2 percentages was most pronounced with increasing doses of CTX. Addition of CTX to everolimus also resulted in an increase in the frequency of pDC, which reached statistical significance at week 4 in both cohort 2 and cohort 5. The expansion cohort (Fig. 3b) confirmed the changes previously noted in patients treated in cohort 2.

**Fig. 4:**
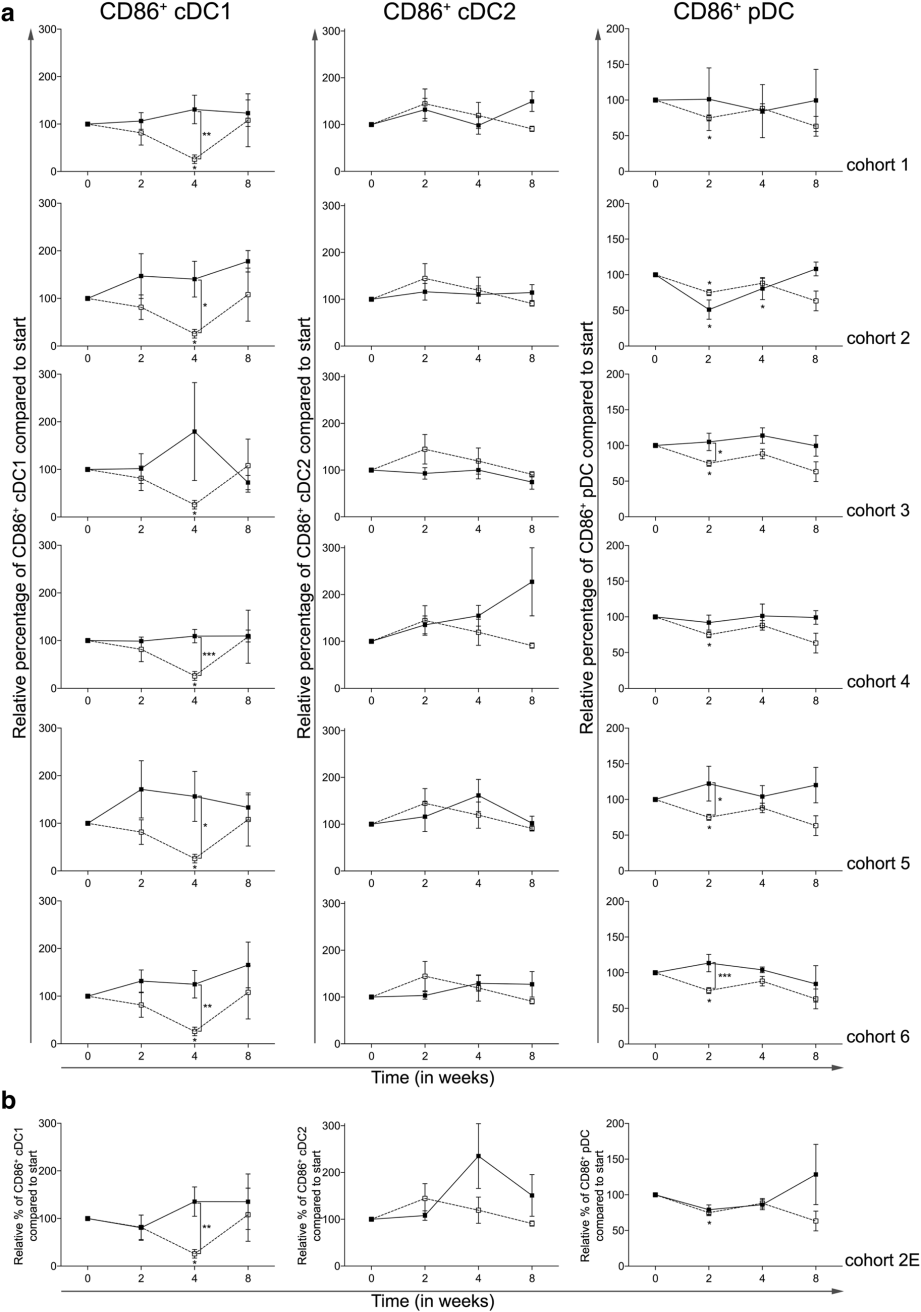
Effect of different dosages and administration schedules of CTX when combined with a fixed dose of 10 mg everolimus on the activation status of three blood DC subsets. **a** Relative MFI of CD86 was determined for the cDC1, cDC2, and pDC subset and shown for the six investigated CTX cohorts (black bullets, black line), compared to cohort 0 (open bullet, dotted line). **b** Relative percentages of the MFI of CD86 on cDC1, cDC2, and pDC shown for the expansion cohort. Means ± SEM are shown

Treatment with everolimus alone resulted in a significant decrease in the expression of CD86 on the cDC1 and pDC subsets, at week 4 and 2, respectively, both with a *p* ≤ 0.05. As shown in Fig. 4a, CTX was capable of reversing this downregulation of CD86 expression on cDC1 reaching statistical significance in all CTX cohorts except cohort 3. While CTX did not result in a significant alteration of CD86 expression on cDC2, an increase in the expression of CD86 on pDC was observed in cohorts 3, 5 and 6 when compared to the patient group treated with everolimus alone (i.e., cohort 0).

Figure 4b shows the changes in MFI of CD86 on the three blood DC subsets in the expansion cohort, again confirming the results previously seen in patients treated in cohort 2.

### Addition of CTX does not result in enhanced NK cell frequencies

**Fig. 5:**
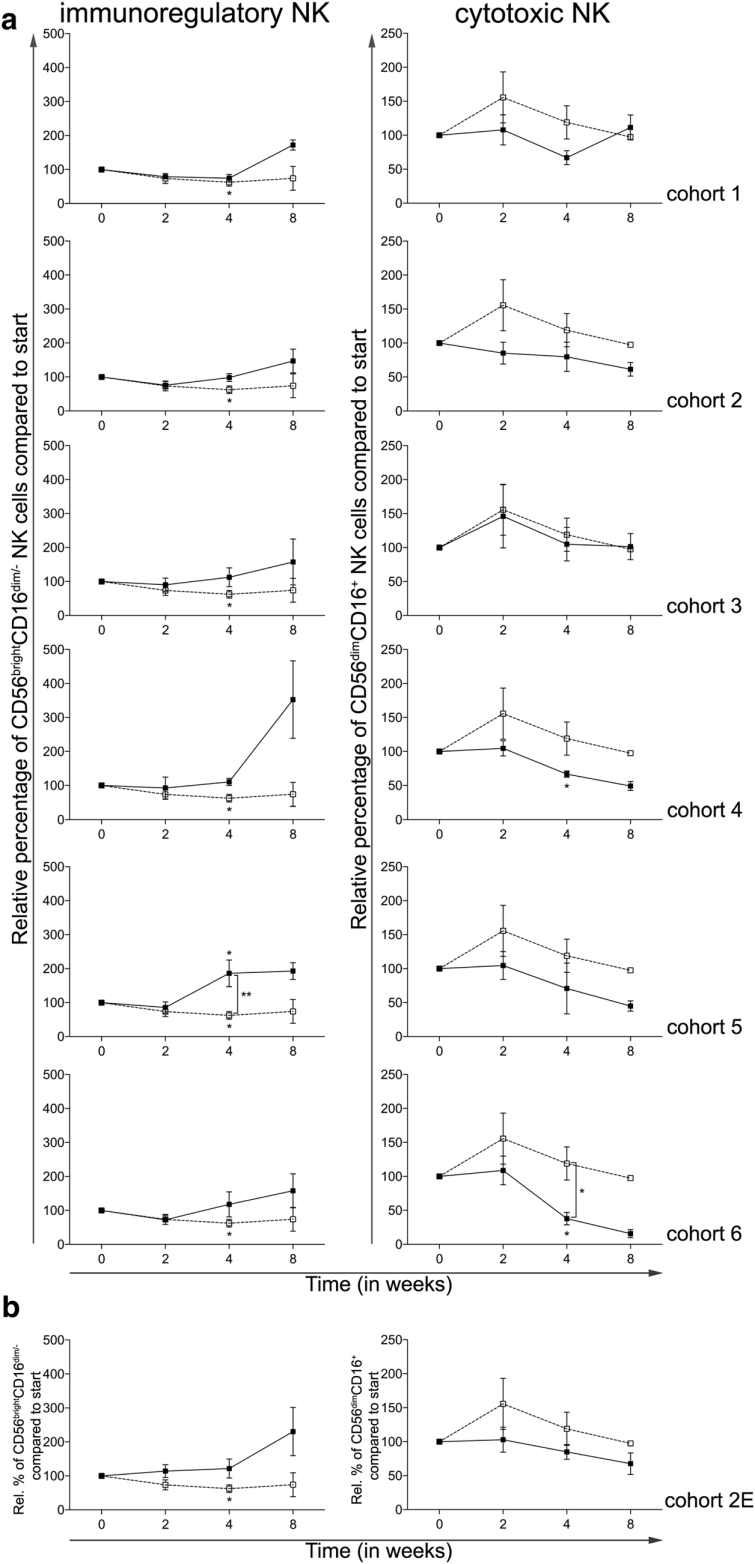
Effect of different dosages and administration schedules of CTX when combined with a fixed dose of 10 mg everolimus on the frequency of NK cells. **a** Relative percentages of immunoregulatory (CD56^bright^CD16^dim/−^) and cytotoxic (CD56^dim^CD16^+^) NK cells are shown for the six investigated CTX cohorts (black bullets, black line), compared to cohort 0 (open bullet, dotted line). **b** Relative percentages of the NK subsets are shown for the expansion cohort. Means ± SEM are shown

A previously published article reported beneficial effects of low-dose metronomic CTX on NK cell function [15]. Two distinct NK cell subsets were monitored, the immunoregulatory CD56^bright^CD16^dim/−^ and the cytotoxic CD56^dim^CD16^+^ subset. As shown in Fig. 5a, 4 weeks of treatment with everolimus alone resulted in a significant reduction in CD56^bright^CD16^dim/−^ NK cells (Fig. 5a, left panels). Addition of CTX resulted in a dose-dependent reversal of this effect with higher doses of CTX actually inducing an increase in the frequency of immunoregulatory NK cells. While treatment with everolimus resulted in a temporary (non-significant) increase in the frequency of CD56^dim^CD16^+^ cytotoxic NK cells, this effect was attenuated by the addition of CTX and actually resulted in a significant decrease in CD56^dim^CD16^+^ NK cells in cohorts 4 and 6 with effects being most striking in cohort 6. Again, the expansion cohort confirmed the earlier observed data in patients treated in cohort 2 (Fig. 5b).

### Overall effect of the addition of continuous once daily oral administration of 50 mg of cyclophosphamide on immune cell populations in patients with mRCC treated with everolimus

**Fig. 6:**
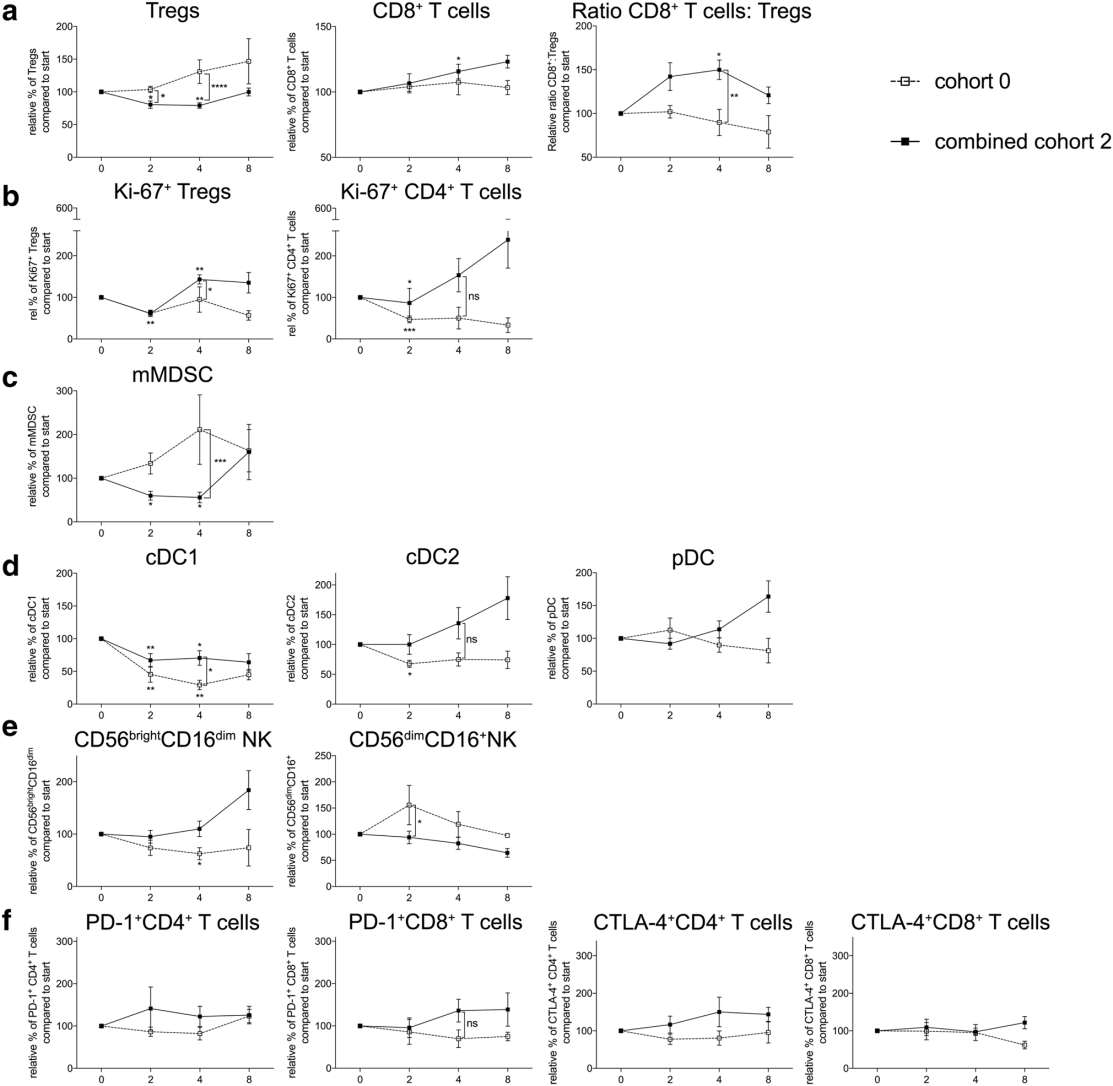
Overview of changes in immune cell subsets in cohort 0 compared to the combined cohort 2 (i.e., cohort 2 and cohort 2E). Patients were treated with 50 mg CTX once daily, combined with 10 mg everolimus once daily. **a** Tregs within CD4^+^ T cells, CD8^+^ T cells within CD3^+^ T cells and the ratio of CD8^+^ T cells versus Tregs are shown. **b** Ki-67 expression in Tregs and CD4^+^ T cells. **c** mMDSC. **d** Blood DC subsets. **e** NK cell subsets. **f** PD-1 and CTLA-4 expression on CD4^+^ and CD8^+^ T cells. Panels show the combined cohort 2 (black bullets, black line) versus cohort 0 (open bullet, dotted line). Means ± SEM are shown

Figure 6 shows the data from patients treated with 10 mg once daily everolimus (i.e., cohort 0) versus all patients treated with the combination of 10 mg once daily everolimus and 50 mg cyclophosphamide in a continuous scheme (i.e., cohort 2 and the expansion cohort, together designated “combined cohort 2”). In this combined cohort, a significant decrease in Tregs was observed and accompanied by an increase in the frequency of CD8^+^ T cells. Together, this resulted in a significant increase in the CD8^+^ T cell:Treg ratio (Fig. 6a). Interestingly, the combined cohort also showed a significant decrease in Ki-67^+^ Tregs at week 2, followed by a significant increase at week 4 compared to week 0. Additionally, a significant difference between the combined cohort and cohort 0 was found at week 4. The Ki-67 expression in CD4^+^ T cells decreased significantly at week 2 and although the Ki-67^+^CD4^+^ T-cell percentages were higher in the combined cohort 2 compared to cohort 0, this difference was not significant (Fig. 6b). Notable was also the observation that whereas mMDSC frequencies increased in patients treated with everolimus alone, the frequency significantly decreased in the combined cohort 2 both at time point 2 as well as 4 weeks (Fig. 6c) with a significant difference between the percentages at time point 4 when comparing the combined cohort to cohort 0. The decrease in cDC1 and cDC2 percentages that was observed with everolimus monotherapy was reversed by adding CTX to the treatment, with a significant difference when comparing to cohort 0 at time point 4 (Fig. 6d). In addition, 50 mg of CTX once daily continuously in combination with everolimus resulted in a reversal of the effect on the immunoregulatory NK cells, however, a decrease in cytotoxic NK cells was observed at time point 2 (*p* < 0.05, Fig. 6e). No significant differences between the combined cohort 2 and cohort 0 were observed for the T-cell activation markers, although there was a notable increase in frequency of CD4^+^CTLA-4^+^ T cells by week 4 (Fig. 6f).

## Discussion

This is the first clinical trial in which several dosages and schedules of metronomic cyclophosphamide in combination with the standard dosage of everolimus were investigated, and where extensive and comprehensive immune monitoring was performed. Our data indicate that while the frequency of Tregs slowly increased during treatment with everolimus alone, the combination of 10 mg everolimus once daily and 50 mg CTX once daily continuously, resulted in a significant decrease of Tregs within 2 weeks of treatment. This decrease persisted up to week 4 returning to baseline levels after 8 weeks of combination treatment. The slight increase in Treg percentages that was observed at 4 and 8 weeks of treatment suggest the reduction in Tregs to be a temporary effect, as was previously also reported in advanced-stage breast cancer patients treated with single-agent 50 mg CTX p.o. daily [20] and which is in line with the observation of an increase in the expression of the proliferation marker Ki-67 in Tregs at time points 4 and 8 weeks. Though this may seem to limit the rationale for combination treatment of CTX and everolimus, Ge et al. [20] reported that transient depletion of Tregs can increase tumor-reactive T-cell numbers implying that even a temporary Treg depletion may sufficiently boost the anti-tumor immune response, by creating a window for T-cell priming against the tumor. In addition, in the combined cohort 2 CD8^+^ T-cell percentages significantly increased and together with the decrease in Treg percentages resulted in an increase in the effector to suppressor ratio (CD8^+^ T cell:Tregs). Since an increased effector to suppressor ratio is associated with improved survival [21–23], this may imply positive effects on the survival of mRCC patients when treated with a combination of everolimus and CTX. Of note, the mechanism behind these reduced cell amounts, e.g., by necrosis or apoptosis, has not been examined.

The combined cohort 2 analysis also revealed a significant decrease in the frequency of mMDSC after 2 and 4 weeks of treatment. As the role of MDSC in the tumor environment is diverse [18] leading to promotion of tumor growth, this mMDSC-depleting effect, though temporary in nature as the decrease in mMDSC did not persist after 4 weeks of combination treatment, could further contribute to improved survival. Of interest, sunitinib has also been reported to decrease the frequency of myeloid suppressor cells [24]. As lenvatinib is not only a TKI directed against the VEGF receptor [25] but additionally inhibits fibroblast growth factor receptor (FGFR) which may also dampen MDSC activity [26], the combination of lenvatinib and everolimus might exert similar or even more pronounced effects on the immune system as the combination of everolimus and CTX.

Previously, we reported that treatment with everolimus alone significantly reduced the frequency of the cDC1 and cDC2 blood DC subsets, while it did not affect the frequency of pDC [27]. Interestingly, we here demonstrate that the addition of any dosage or scheme of CTX could reverse these everolimus-induced alterations in the frequency of cDC1 and cDC2. Furthermore, CTX was also able to increase the activation of at least two of the three blood DC subsets, the cDC1 and pDC subset. While cohort 2 already showed beneficial effects on the blood DC subsets, effects were even more pronounced when higher doses of CTX were used.

As Ghiringhelli et al. [15] reported beneficial effects of CTX treatment on NK and T cell effector functions, we were interested in the effects of the combination of everolimus and CTX on both cell subsets. We found that adding CTX to everolimus could reverse the effects of everolimus monotherapy on the immunoregulatory CD56^bright^CD16^dim/−^ NK cell subset as well as on the cytotoxic CD56^dim^CD16^+^ NK cell population, overall resulting in an increase in the frequency of immunoregulatory NK cells and a decrease in the frequency of cytotoxic NK cells with combination therapy. For both NK cell subsets, functional analyses were not performed, and therefore it remains impossible to determine whether these subsets are also functionally compromised as previously reported [28]. For both the CD4^+^ and CD8^+^ T cell subset the expression of PD-1 and CTLA-4 was determined. Though not significant, a minor increase in expression of PD-1 and CTLA-4 could be noted, perhaps suggesting an increase in tumor-specific effector T cells and a window for combination therapy with immune checkpoint inhibitors targeting CTLA-4 and/or PD-1 [29].

In conclusion, we performed a phase 1 study in patients with mRCC treated with everolimus alone and the combination of everolimus and different doses and administration schedules of CTX and here report on the comprehensive immunomonitoring that was performed in these patients. The predefined goal of the study, i.e., to identify the dose and schedule of CTX that when combined with everolimus would result in optimal and selective depletion of Tregs, was achieved with a once daily continuous oral dose of 50 mg CTX. Addition of this dose of CTX to everolimus, resulted in depletion of Tregs, a sustained increase in CD8^+^ T cells with an increase in the effector to suppressor ratio. Furthermore, this combination therapy resulted in a depletion of mMDSC, while negative effects of monotherapy with everolimus on blood DC subsets were counteracted. All together, these observed changes in various immune cell populations may result in increased antitumor immunity and improved survival of patients with mRCC, which is currently further investigated in a phase 2 clinical trial [16].

## Electronic supplementary material

Below is the link to the electronic supplementary material.

Supplementary material 1 (PDF 2141 KB)

## Abbreviations

APC: Allophycocyanin
CCMO: Committee on Research Involving Human Subjects
cDC: Conventional DC
CTX: Cyclophosphamide
FGFR: Fibroblast growth factor receptor
ICH: International Conference on Harmonization
mTOR: Mammalian target of rapamycin
mRCC: Metastatic RCC
mMDSC: Monocytic MDSC
pDC: Plasmacytoid DC
PFS: Progression-free survival
Tregs: Regulatory T cells
RCC: Renal cell carcinoma
WIN-O: The Netherlands Working Group on Immunotherapy of Oncology
