# Supplementary material for: The effect of everolimus and low-dose cyclophosphamide on immune cell subsets in patients with metastatic renal cell carcinoma: results from a phase I clinical trial
Source: Cancer Immunol Immunother. 2019 Jan 17;68(3):503–15. doi: 10.1007/s00262-018-2288-8 (PMC6426984; doi:10.1007/s00262-018-2288-8)
Supplement: Supplementary file 1 — Supplementary material 1 (PDF 2141 KB) [file 262_2018_2288_MOESM1_ESM.pdf]

Supplementary Fig. 1

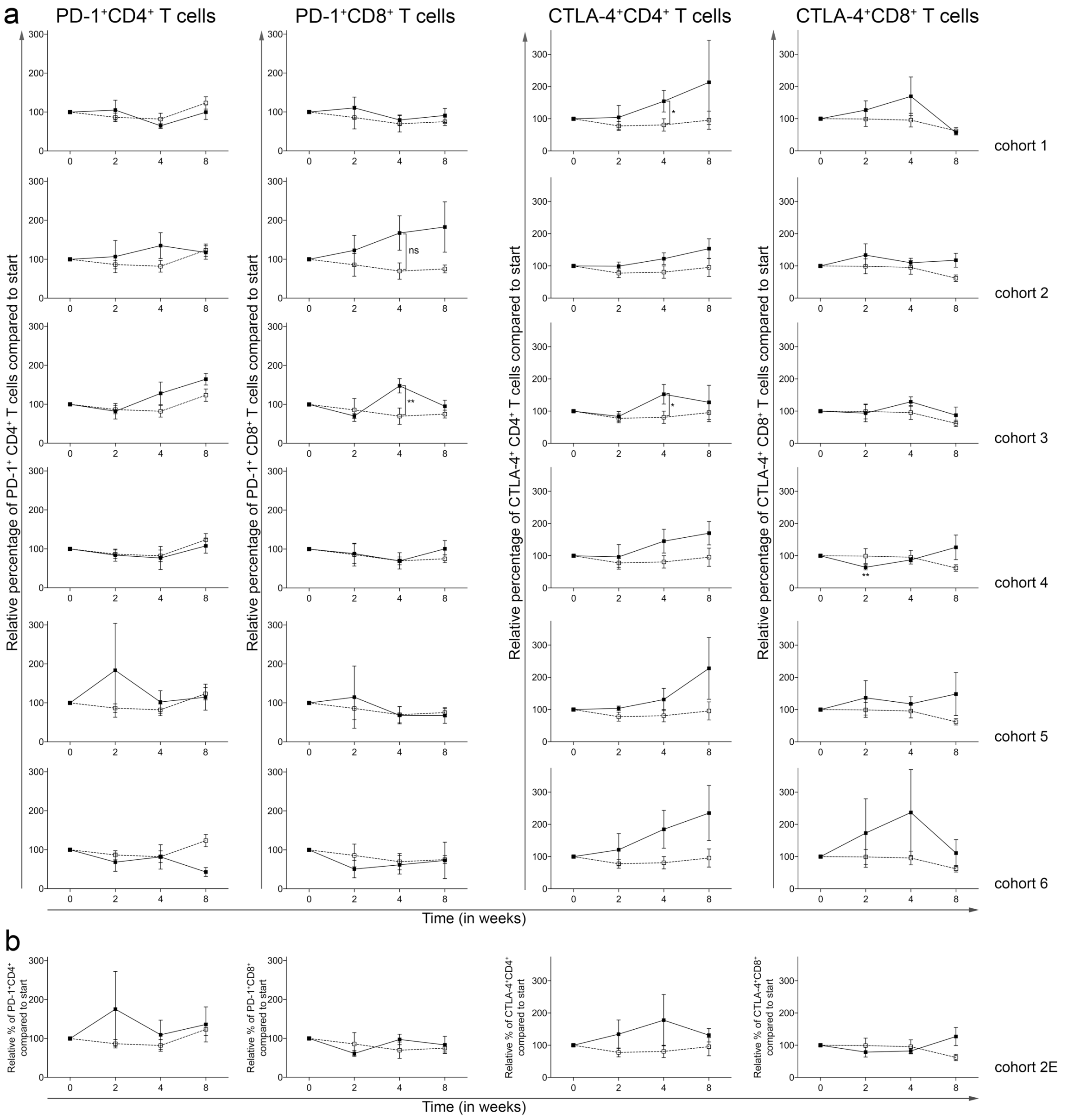

Effect of different dosages and administration schedules of CTX when combined with a fixed dose of 10 mg everolimus on the frequency of PD-1 and CTLA-4 expression on $CD4^+$ and $CD8^+$ T cells. a. Relative percentages (to start) are shown for the 6 investigated CTX cohorts (black bullets. black line). compared to cohort 0 (open bullet. dotted line). b. Relative percentages of the subsets are shown for the expansion cohort. Means ± SEM are shown.

Supplementary Fig 2.

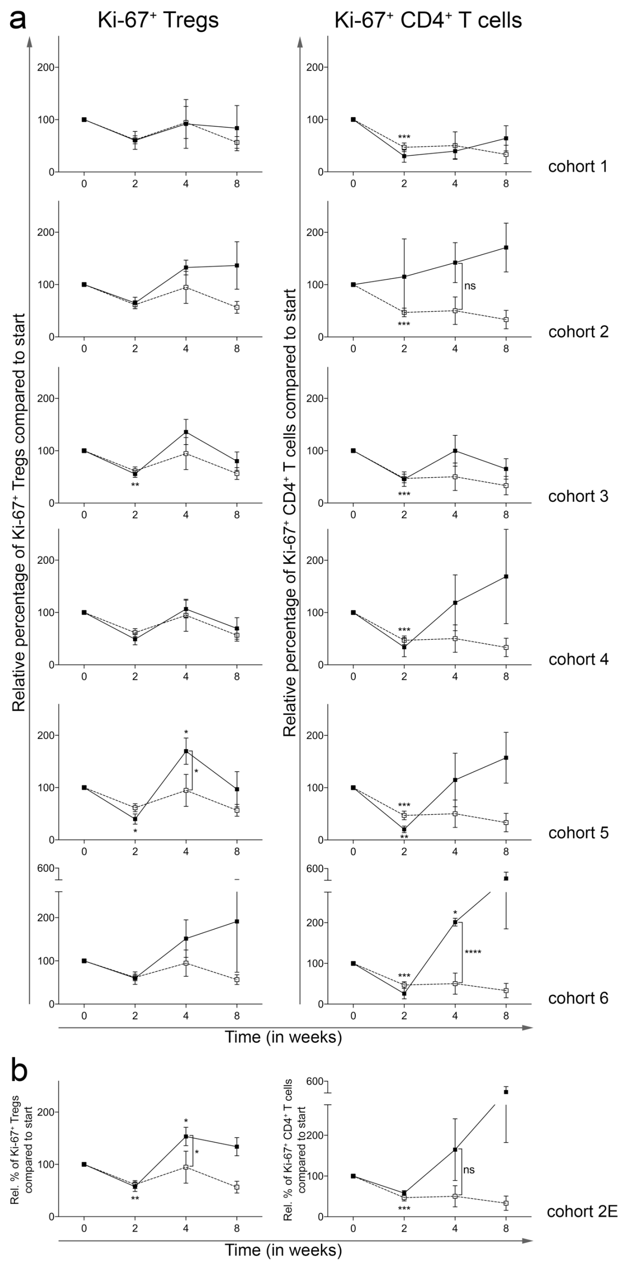

Effect of different dosages and administration schedules of CTX when combined with a fixed dose of 10 mg everolimus on the frequency of Ki-67 expression in Tregs and $CD4^+$ T cells. a. Relative percentages of Ki-67$^+$Tregs and Ki-67$^+$CD4$^+$ T cells (relative to start) are shown for the 6 investigated CTX cohorts (black bullets. black line). compared to cohort 0 (open bullet. dotted line). b. Relative percentages of Ki-67$^+$Tregs and Ki-67$^+$CD4$^+$ T cells are shown for the expansion cohort. Means ± SEM are shown.

Supplementary Fig 3.

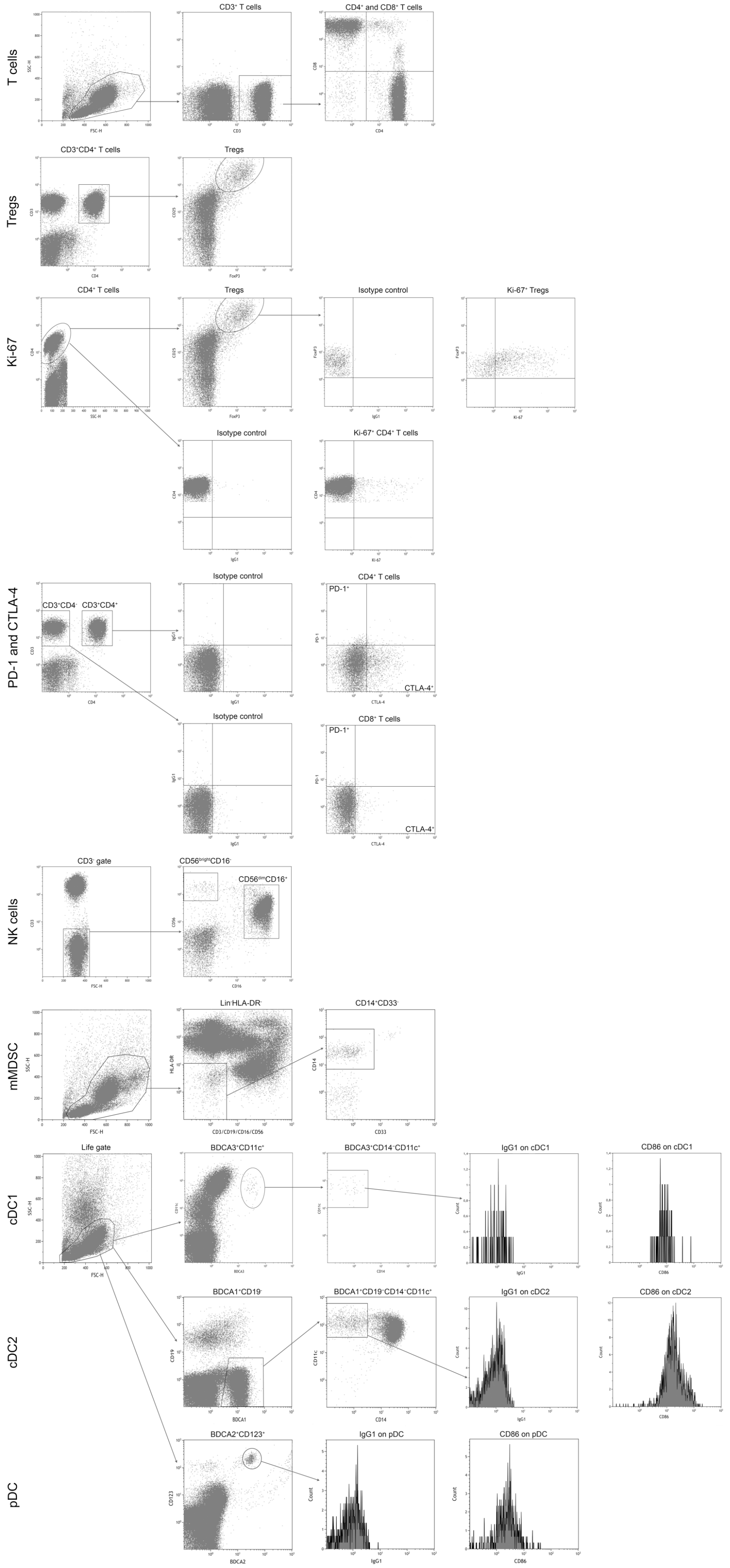

Gating strategy of all described subsets.

Supplementary table 1. Mean CD3+ and CD4+ T cell percentages and absolute numbers per cohort and per time point with SEM.

CD3+ T cell percentages within PBMC (±SEM)

	Time point (in weeks)			
	Week 0	Week 2	Week 4	Week 8
Cohort 0	50.0 (3.3)	55.9 (4.0)	49.1 (3.0)	42.6 (8.3)**
Cohort 1	44.0 (6.4)	49.0 (4.2)	40.0 (6.1)*	39.1 (11.2)**
Cohort 2	41.5 (7.9)	51.4 (8.1)	40.2 (5.7)	32.2 (6.3)
Cohort 3	44.1 (5.6)	53.4 (4.7)	41.2 (4.4)	39.0 (3.4)
Cohort 4	26.4 (4.4)	37.2 (5.9)	36.1 (7.3)	35.7 (8.0)
Cohort 5	44.2 (5.9)	61.4 (3.5)	57.6 (6.2)	45.5 (5.6)*
Cohort 6	37.4 (2.7)*	44.7 (5.9)*	31.2 (5.2)*	22.8 (5.3)**
Cohort 2E	58.4 (4.4)	62.0 (4.3)	59.7 (8.3)	60.7 (7.0)*

*n= 4 **n=3 instead of n=5

Absolute CD3+ T cell numbers/L peripheral blood (±SEM)

	Time point (in weeks)			
	Week 0	Week 2	Week 4	Week 8
Cohort 0	1.3E+09 (3.1E+08)	8.3E+08 (1.7E+08)	1.1E+09 (2.4E+08)*	7.7E+08 (2.9E+08)**
Cohort 1	8.0E+08 (1.2E+08)*	9.3E+08 (5.7E+07)*	5.7E+08 (1.2E+08)**	5.8E+08 (1.3E+08)**
Cohort 2	8.4E+08 (2.4E+08)*	7.1E+08 (2.4E+08)*	7.2E+08 (1.9E+08)	5.4E+08 (1.7E+08)
Cohort 3	1.4E+09 (3.0E+08)**	1.1E+09 (1.1E+08)**	9.9E+08 (3.6E+08)**	9.2E+08 (4.3E+08)**
Cohort 4	5.2E+08 (1.3E+08)	4.9E+08 (1.1E+08)	4.2E+08 (1.2E+08)	3.1E+08 (8.8E+07)
Cohort 5	6.2E+08 (1.1E+08)	5.3E+08 (8.4E+07)**	6.5E+08 (1.3E+08)	4.1E+08 (4.2E+07)*
Cohort 6	8.0E+08 (1.4E+08)	6.3E+08 (1.3E+08)	3.5E+08 (1.1E+08)	2.0E+08 (1.4E+08)
Cohort 2E	1.3E+09 (3.7E+08)*	9.2E+08 (1.6E+08)	9.0E+08 (1.9E+08)	9.1E+08 (2.7E+08)*

*n= 4 **n=3 instead of n=5

CD4+ T cell percentages within CD3 (±SEM)

	Time point (in weeks)			
	Week 0	Week 2	Week 4	Week 8
Cohort 0	53.7 (6.0)	51.5 (5.6)	53.1 (5.9)	50.7 (7.4)**
Cohort 1	57.2 (2.6)	55.9 (6.0)	59.9 (4.9)*	54.7 (4.6)**
Cohort 2	66.1 (9.9)	67.1 (8.7)	65.4 (9.2)	62.1 (8.9)
Cohort 3	49.3 (8.8)	48.6 (8.8)	50.9 (7.3)	44.0 (6.9)
Cohort 4	54.7 (5.1)	54.3 (6.1)	44.1 (10.8)	52.6 (8.6)
Cohort 5	55.1 (5.6)	52.6 (5.4)	54.4 (5.9)	41.3 (10.1)*
Cohort 6	71.9 (7.9)*	67.9 (10.1)*	68.8 (12.6)*	60.8 (9.5)**
Cohort 2E	62.7 (5.3)	60.8 (5.8)	53.2 (8.8)	50.3 (7.1)*

*n= 4 **n=3 instead of n=5

Absolute CD4+ T cell numbers/L peripheral blood (±SEM)

	Time point (in weeks)			
	Week 0	Week 2	Week 4	Week 8
Cohort 0	6.6E+08 (1.5E+08)	4.1E+08 (6.7E+07)	5.1E+08 (1.1E+08)*	3.8E+08 (1.5E+08)**
Cohort 1	4.7E+08 (8.4E+07)*	5.6E+08 (8.2E+07)*	3.6E+08 (6.7E+07)**	3.1E+08 (5.9E+07)**
Cohort 2	5.2E+08 (1.8E+08)*	4.8E+08 (1.7E+08)*	4.8E+08 (1.4E+08)*	3.2E+08 (1.0E+08)*
Cohort 3	6.9E+08 (1.2E+08)**	5.2E+08 (8.1E+07)**	4.9E+08 (1.1E+08)**	3.7E+08 (1.3E+08)**
Cohort 4	2.7E+08 (5.7E+07)	2.4E+08 (2.7E+07)	2.2E+08 (5.2E+07)	1.6E+08 (3.8E+07
Cohort 5	3.4E+08 (7.4E+07)	2.8E+08 (6.0E+07)**	3.5E+08 (7.4E+07)	1.9E+08 (1.6E+07)*
Cohort 6	5.6E+08 (1.1E+08)	4.3E+08 (1.1E+08)	2.3E+08 (6.2E+07)	1.1E+08 (6.6E+07)
Cohort 2E	8.3E+08 (2.7E+08)*	5.3E+08 (6.4E+07)	4.3E+08 (6.0E+07)	4.9E+08 (2.2E+08)*

*n= 4 **n=3 instead of n=5

Supplementary table 2. Mean percentages and absolute numbers of PD-1 and CTLA-4 on $CD4^+$ and $CD8^+$ T cells.

Percentage of cells (±SEM) at baseline

	$PD\text{-}1^+CD4^+$	$PD\text{-}1^+CD8^+$	$CTLA\text{-}4^+CD4^+$	$CTLA\text{-}4^+CD8^+$
Cohort 0	6.1 (3.9)	4.8 (1.6)	8.6 (1.1)	3.3 (1.0)
Cohort 1	1.8 (0.3)	1.7 (0.3)	3.5 (0.6)	5.3 (2.0)
Cohort 2	1.6 (0.6)	2.1 (0.4)	7.4 (1.6)	3.4 (0.4)
Cohort 3	2.2 (1.0)	1.8 (0.4)	8.2 (1.9)	8.2 (1.9)
Cohort 4	1.2 (0.1)	1.6 (0.1)	8.1 (1.4)	3.4 (0.6)
Cohort 5	0.9 (0.3)	1.1 (0.3)	6.4 (0.7)	2.0 (0.3)
Cohort 6	1.4 (0.2)*	4.0 (1.6)*	6.2 (1.0)*	2.2 (0.6)*
Cohort 2E	1.0 (0.4)	0.8 (0.4)	4.6 (0.8)	1.5 (0.5)

*n= 4 instead of n=5

Absolute numbers of cells/L peripheral blood (±SEM) at baseline

	$PD\text{-}1^+CD4^+$	$PD\text{-}1^+CD8^+$	$CTLA\text{-}4^+CD4^+$	$CTLA\text{-}4^+CD8^+$
Cohort 0	2.5E+07<br>(1.1E+07)	1.9E+07<br>(4.5E+06)	5.8E+07<br>(1.5E+07)	1.7E+07<br>(7.9E+06)
Cohort 1	7.8E+06<br>(1.5E+06)*	4.7E+06<br>(9.7E+05)*	1.6E+07<br>(3.6E+06)*	6.0E+06<br>(7.0E+05)*
Cohort 2	6.1E+06<br>(1.7E+06)*	4.0E+06<br>(1.5E+06)*	7.9E+06<br>(2.5E+06)*	3.4E+07<br>(5.7E+06)*
Cohort 3	2.0E+07<br>(1.3E+07)**	8.4E+06<br>(2.3E+06)**	4.3E+07<br>(1.5E+07)**	1.6E+07<br>(3.7E+06)**
Cohort 4	3.1E+06<br>5.5E+05	3.0E+06<br>7.7E+05	2.2E+07<br>5.2E+06	5.9E+06<br>1.2E+06
Cohort 5	3.2E+06<br>1.5E+06	1.9E+06<br>5.0E+05	2.3E+07<br>7.4E+06	4.0E+06<br>8.8E+05
Cohort 6	7.6E+06<br>(2.0E+06)*	4.6E+06<br>(7.0E+05)*	3.5E+07<br>(8.0E+06)*	3.7E+06<br>(1.1E+06)*
Cohort 2E	5.0E+06<br>(1.8E+06)*	4.3E+06<br>(2.6E+06)*	4.4E+07<br>(2.0E+07)*	6.4E+06<br>(3.5E+06)*

*n= 4 **n=3 instead of n=5
